# Nanoparticles for efficient drug delivery and drug resistance in glioma: New perspectives

**DOI:** 10.1111/cns.14715

**Published:** 2024-05-06

**Authors:** Jiyuan Liu, Fan Yang, Jinqu Hu, Xiuchun Zhang

**Affiliations:** ^1^ Department of Neurosurgery the First Hospital of China Medical University Shenyang China; ^2^ Department of Cardiology the Fourth Affiliated Hospital of China Medical University Shenyang China; ^3^ Department of Neurology the First Hospital of China Medical University Shenyang China

**Keywords:** chemotherapy resistance, glioma, nanoparticles, temozolomide (TMZ), therapeutic target

## Abstract

Gliomas are the most common primary tumors of the central nervous system, with glioblastoma multiforme (GBM) having the highest incidence, and their therapeutic efficacy depends primarily on the extent of surgical resection and the efficacy of postoperative chemotherapy. The role of the intracranial blood–brain barrier and the occurrence of the drug‐resistant gene O6‐methylguanine‐DNA methyltransferase have greatly limited the efficacy of chemotherapeutic agents in patients with GBM and made it difficult to achieve the expected clinical response. In recent years, the rapid development of nanotechnology has brought new hope for the treatment of tumors. Nanoparticles (NPs) have shown great potential in tumor therapy due to their unique properties such as light, heat, electromagnetic effects, and passive targeting. Furthermore, NPs can effectively load chemotherapeutic drugs, significantly reduce the side effects of chemotherapeutic drugs, and improve chemotherapeutic efficacy, showing great potential in the chemotherapy of glioma. In this article, we reviewed the mechanisms of glioma drug resistance, the physicochemical properties of NPs, and recent advances in NPs in glioma chemotherapy resistance. We aimed to provide new perspectives on the clinical treatment of glioma.

## Graphical abstract

The key players in the initiation and progression of glioma. Studies have shown that angiogenesis, blood–brain barrier dysfunction, and poor lymphatic drainage are major factors in glioma development. In addition, other factors such as mutations in genes such as IDHI, TP53, and RB1, as well as PTEN and INK4 deletions, and CDK4, VEGF, and EGFR amplification also play an important role in glioma formation.

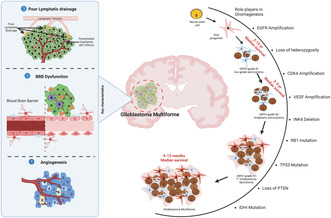

## 1 INTRODUCTION

Gliomas are the most common primary malignant tumors within brain parenchyma in adults, accounting for approximately 2% of all cancers.^1^, ^2^ Based on histologic features, gliomas are classified into various types, including astrocytomas, glioblastoma multiforme (GBM), oligodendrogliomas, and ventriculomeningiomas.^3^ Of these, WHO grade IV GBM is the most common and aggressive primary brain tumor.^4^ The exact cause of glioma is still unknown, and many researchers believe that it may be related to congenital genetic factors such as familial predisposition and susceptibility genes, acquired environmental factors such as ionizing radiation (X‐rays, gamma rays, etc.), chemicals (organic solvents such as benzene), some drugs, and poor lifestyle habits such as smoking and drinking.^5^, ^6^, ^7^ In the treatment of gliomas, surgical resection with maximum safety, radiation therapy, and chemotherapy with temozolomide (TMZ) are the main recommendations for patients in good physical condition.^8^, ^9^ The goals of surgical treatment are to clarify the pathologic diagnosis, reduce tumor volume, decrease tumor cell count, improve symptoms, relieve hypercapnic pressure symptoms, prolong life, and create time for other subsequent comprehensive treatments. However, due to the proliferative nature of gliomas, they cannot be completely removed by surgery and are prone to recurrence after surgery.^10^ Radiation therapy is one of the conventional treatment modalities for glioma, but its efficacy has not been consistently evaluated. Whole‐brain irradiation does not significantly improve prognosis, but local irradiation is at least as effective as whole‐brain irradiation. Therefore, to avoid irreversible damage to normal brain tissue caused by whole‐brain irradiation, irradiation is performed primarily in the tumor area.^11^ As a rule, chemotherapy is used to treat malignant tumors, but chemotherapeutic agents have limitations such as the blood–brain barrier (BBB) and toxic side effects of the drugs.^12^, ^13^, ^14^ For low‐grade gliomas, cure criteria can be achieved after total surgical resection, with a median survival of up to 50 years. In contrast, high‐grade gliomas are prone to recurrence, have a poor prognosis, and a short survival time even after surgical treatment.^15^

Chemotherapy resistance is a common challenge in tumor therapy because tumor cells acquire resistance to chemotherapeutic drugs and are unable to effectively kill tumor cells.^16^, ^17^ This phenomenon is usually caused by biochemical mechanisms within the tumor cells, such as drug efflux, drug catabolism, DNA repair.^18^, ^19^, ^20^, ^21^, ^22^, ^23^ In the field of oncology, NPs have been extensively studied as drug carriers to overcome the problem of drug resistance faced by conventional chemotherapeutic agents.^24^, ^25^ NPs as drug carriers have the following advantages. First, NPs can be used efficiently for drug delivery, encapsulating chemotherapeutic agents and ensuring high drug concentrations are distributed to tumor sites to improve therapeutic efficacy.^22^, ^26^ NPs can also carry multiple drugs and can overcome the problem of drug resistance through combination therapy.^27^, ^28^, ^29^, ^30^

Overall, this article outlined the mechanisms of chemotherapeutic drug resistance in glioma, the physicochemical properties of NPs, and recent advances in NPs in glioma chemotherapeutic drug resistance. We aimed to provide new perspectives on the clinical treatment of glioma.

## 2 BLOOD–BRAIN BARRIER

### 2.1 The overview of BBB

**FIGURE 1:**
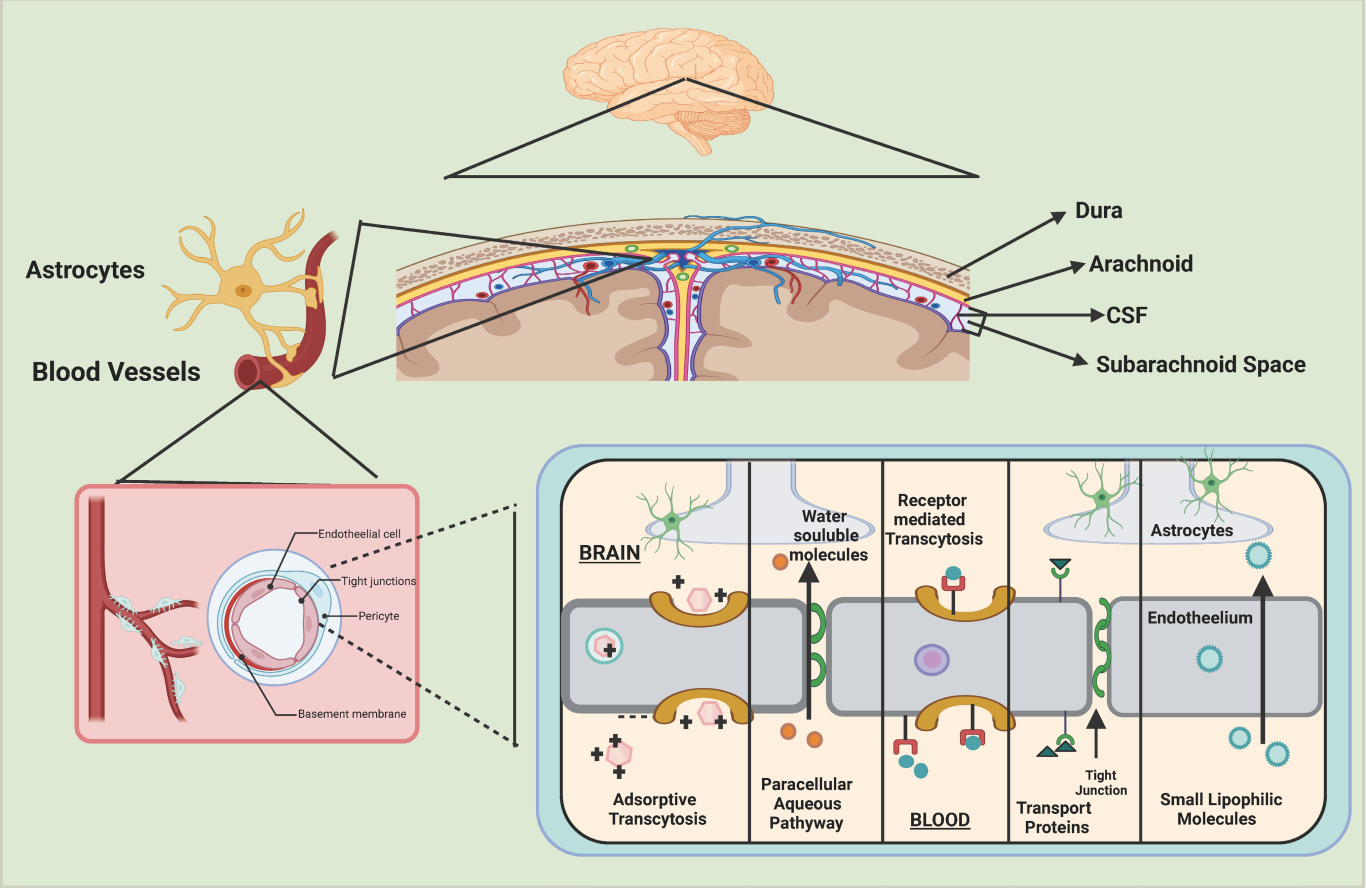
The illustrative representation of the blood–brain barrier (BBB) and transport mechanisms across the BBB. The BBB consists mainly of blood vessels and endothelial cells (EC), whose tight junctions are surrounded by astrocyte end‐feet and pericytes. Because of the presence of tightly woven endothelial cells covering cerebral capillaries, the BBB effectively restricts the entry of unwanted molecules into the brain cells and thus allows access to vital nutrients for normal metabolism. Except for specific transport channels, 100% of biomolecules and 98% of small molecules cannot cross the BBB to enter the brain by passive diffusion mechanisms, which severely limits the efficacy of drug therapy for brain diseases. Therefore, energy‐dependent pathways including transporter proteins, receptor‐mediated transport and adsorptive transport must be used for transport across the BBB.

An important structural difference between the brain and the peripheral vasculature is the BBB, which tightly controls the transport of molecules between the brain and bloodstream and maintains the balance of the CNS internal environment.^31^, ^32^ The BBB is a special type of brain microvascular endothelium and its tight junctions (TJs) The BBB is a dense diffusion barrier formed by a special type of cerebral microvascular endothelium and its TJs that severely limits the paracellular transport of hydrophilic molecules, allowing only a few lipophilic substances and respiratory gases to diffuse freely.^33^, ^34^ Among them, occludin, claudin, and ZO are important proteins that comprise TJs.^35^ TJs between BBB endothelial cells confer high endothelial cell resistance and low cell bypass permeability. In addition, the cytoskeleton, consisting of actin microfilaments, microtubules, and intermediate fibers, is involved in maintaining the integrity of endothelial intercellular junctions. The actin microfilament system is specifically associated with various membrane adhesion proteins such as calreticulin, occludin, protein complexes, and functional intercellular proteins, and its structure is closely related to the endothelial cell tension formed by the phosphorylation of myosin light chains and actin stress fibers.^36^, ^37^ Microtubules are involved in the rapid self‐organization of actin microfilaments and focal adhesions, isometric cell contraction, and facilitation of leukocyte migration across the endothelium. Intermediate fibers are dynamically altered during reorganization of actin microfilaments and microtubules, but their specific mechanisms of action in cytoskeletal changes are still unknown. Furthermore, adherens junctions (AJs) are usually intermingled with TJs; in AJs, the endothelium‐specific membrane protein VE‐calmodulin is linked to the cytoskeleton via connexins of the armadillo protein family. In the BBB structure, β‐connexin and χ‐connexins are essential for the regulation of AJ function.^38^ Caveolin‐1‐induced downregulation of ZO‐1 and occludin is associated with changes in AJs proteins, VE‐calmodulin, and β‐connexin, and may be associated with enhanced stimulation of monocyte epithelial migration by chemokine ligand 2.^39^ Thus, changes in AJs proteins may increase the permeability of BBB cell bypass and facilitate leukocyte entry into the CNS. Ve‐calmodulin is an essential component for the functional integrity of quiescent and reconstituted neovessels. The BBB is a key component for normal brain function and central nervous system. Studies have shown that the BBB maintains a balance between normal brain function and the internal environment of the CNS. Except for specialized transport channels, 100% of biopolymer drugs and 98% of small molecule drugs cannot pass through the BBB into the brain via passive diffusion mechanisms, severely limiting the efficacy of pharmacological treatments for brain diseases. Therefore, how to effectively improve the efficiency of transport of therapeutic drugs into the brain is an important problem to be solved in the current pharmacotherapy for brain diseases. The illustrative representation of the BBB and transport mechanisms across the BBB is displayed in Figure 1.

### 2.2 The role of BBB in glioma

**FIGURE 2:**
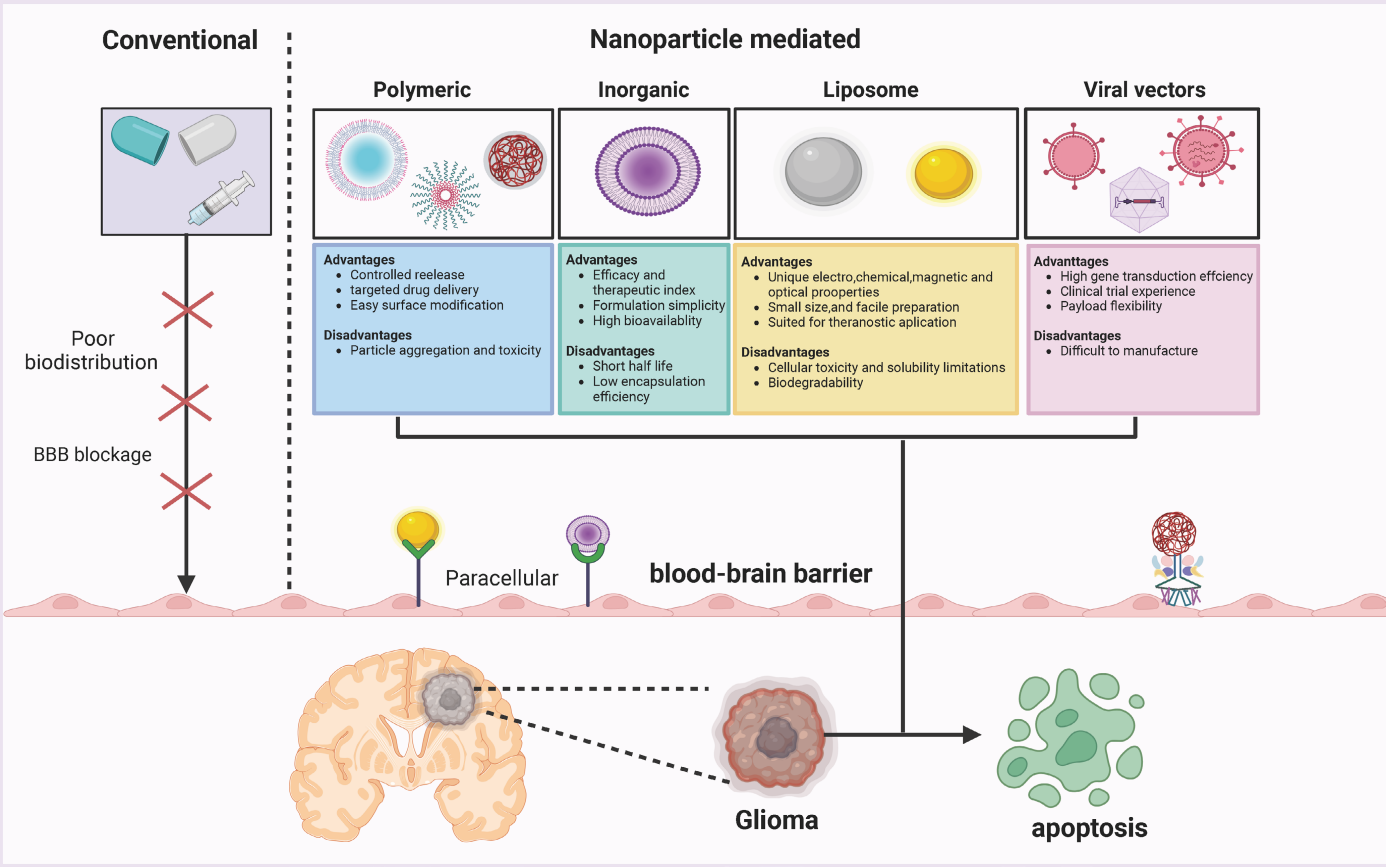
The different methods of drug delivery across the blood–brain barrier (BBB) in GBM. Studies have shown that various nanocarriers such as polymeric, inorganic, liposome, and viral vectors can be used to deliver therapeutic agents between the BBB and targeted brain tumors.

Furthermore, the BBB may play an important role in the development and treatment of gliomas. Physiologically, the BBB is a biofilm barrier that protects and maintains CNS function.^31^, ^40^ Pathological conditions usually involve pathological “disruption” of BBB function, with disruption of TJs structure, decreased expression of TJ proteins, and increased BBB permeability.^12^, ^41^ On the one hand, pathological “disruption” of BBB function leads to disruption of the balance of the CNS internal environment, leakage of toxic substances from the bloodstream into the CNS, formation of cellular infiltrates, abnormal molecular transport and clearance, accelerated disease progression, and poor disease prognosis. On the other hand, the pathological “open window” of the BBB provides a paracellular pathway for drug transport into the brain, significantly increasing the efficiency of therapeutic drug release in the brain. Therefore, based on the structural features of the BBB, designing appropriate brain drug release systems that facilitate drug release into the brain may provide new ideas and opportunities for the effective treatment of brain diseases. The different methods of drug delivery across the BBB in glioma are shown in Figure 2.

## 3 ADVANCES IN CHEMOTHERAPY FOR GLIOMA

Nitrosourea agents such as lomustine, simustine, formostine, carmustine, nimustine, and PCV (procarbazine + lomustine + vincristine) can cross the BBB and were first used for chemotherapy of gliomas.^42^, ^43^ Combination therapy consisting of cytotoxic chemotherapeutic agents such as teniposide, etoposide, isocyclophosphamide, cisplatin, and carboplatin has also been used occasionally.^2^, ^44^, ^45^ Regimens are also used occasionally but the efficacy of these chemotherapy regimens is limited and their cytotoxic effects are significant.

### 3.1 Mechanisms of chemotherapy resistance in glioma

Drug resistance is the inability or decreased sensitivity of tumor cells to antitumor drugs that normally kill tumor cells. The development of tumor resistance is a major cause of chemotherapy failure; the BBB and the blood–cerebrospinal fluid barrier prevent the entry of high‐molecular‐weight antitumor drugs into tumor cells. The blood–tumor barrier (BTB), on the other hand, is an additional barrier that prevents drugs from entering tumor cells via interactions between membrane transporter proteins overexpressed in the endothelial cells that comprise the BTB and extracellular drug molecules in tumor cells. Resistance of some tumors to the nitrosourea‐based chemotherapy drugs ACNU and cisplatin is achieved in part by this mechanism.^46^ Increased efflux of chemotherapeutic drugs from tumor cells is mediated primarily through the ATP‐binding cassette (ABC) superfamily of transporter proteins in the tumor cell membrane, which, depending on the chemical energy of ATP, actively transport various antitumor drugs out of the cell, resulting in tumor cell resistance to multiple drugs appear.^47^, ^48^ The ABC superfamily of transporter proteins consists of at least 50 proteins, each member of which is highly resistant to various drugs; the ABC transporter superfamily consists of at least 50 proteins, each with a highly conserved ATP‐binding subunits. These transporter proteins are generally composed of four structural domains, two of which are hydrophobic domains (each with six transmembrane structures, forming a transmembrane transport pathway), two of which are ATP‐binding domains and a catalytic domain, which are responsible for providing chemical energy for the transmembrane transport of membrane substances by the membrane transporter proteins.^49^, ^50^ The two are responsible for supplying chemical energy to the human ABC transporter proteins are divided into seven subfamilies, named ABCA through ABCG in sequence, and are mainly distributed in organs with secretory and excretory functions, protecting the body from external toxins and drugs.^51^, ^52^ At least 12 transporter proteins of the ABC transporter family have been shown to be associated with tumor cell drug resistance, facilitation of drug catabolism by tumor cells. Accelerated catabolism of drugs by tumor cells results in lower drug concentrations in tumor cells and reduced drug killing efficacy.^53^, ^54^ Intrinsic molecular mutations in tumor cells: this increases the ability of tumor cells to repair DNA damage and decreases apoptotic activity, thereby reducing the killing ability of antitumor drugs to some extent.

In addition, drug resistance in gliomas may be related to mechanisms such as heterogeneity, hypermutation, immune evasion, and selective splicing for tumor activation. Heterogeneity refers to the presence of cells with different characteristics in a tumor cell population, some of which are more resistant to antitumor drugs.^44^, ^55^ Heterogeneity in gliomas can be elucidated together in terms of both clonal evolution and tumor stem cells.^4^, ^56^, ^57^ According to the theory of clonal evolution, the accumulation of successive mutations within a cell leads to its extracellular proliferation, and these extracellular proliferations grow abnormally as a result of selection pressure from the microenvironment. Hypermutation is the mutation of specific genes in tumor cells, resulting in reduced sensitivity to drugs.^58^, ^59^ Immune evasion is the avoidance of tumor cells being recognized and attacked by the immune system, for example by suppressing the immune response. Gliomas reside in the brain and were previously thought to be immunologically privileged and immune surveillance spared, but recent studies have shown that the brain is an active regulatory point of immune surveillance.^57^, ^60^ Without disruption of the BBB, the infiltration of peripheral immune cells into gliomas is very limited. However, when the BBB is disrupted by tumor growth or inflammation, gliomas are infiltrated by immunosuppressive cells. The immunosuppressive microenvironment of gliomas is a combination of immunosuppressive cytokines and chemokines, immune cells, T/B regulatory cells, tumor‐associated macrophages and bone marrow derived suppressor cells. Under conditions of an immunosuppressive microenvironment, the expression of immune checkpoint receptors (including CTLA4 and PD‐1) is dramatically increased. These receptors are expressed on the surface of T cells and play a negative regulatory role in T cell activation, avoiding immune overactivation and inducing immune escape and drug resistance.^61^, ^62^ Selective splicing is the process of generating different mRNA splice isoforms from mRNA precursors by different splicing methods and can result in tumor drug resistance.^63^, ^64^ This may be related to the level of RNA‐binding proteins, mutations in splice sites, or regulatory elements. Small nucleolar ribonucleoprotein polypeptide B (SNRPB) is a major component of the sialosome and one of the major effectors of cell viability, proliferation, and apoptosis.^65^

### 3.2 Current drugs used to treat glioma and their resistance mechanisms

#### 3.2.1 Temozolomide

**FIGURE 3:**
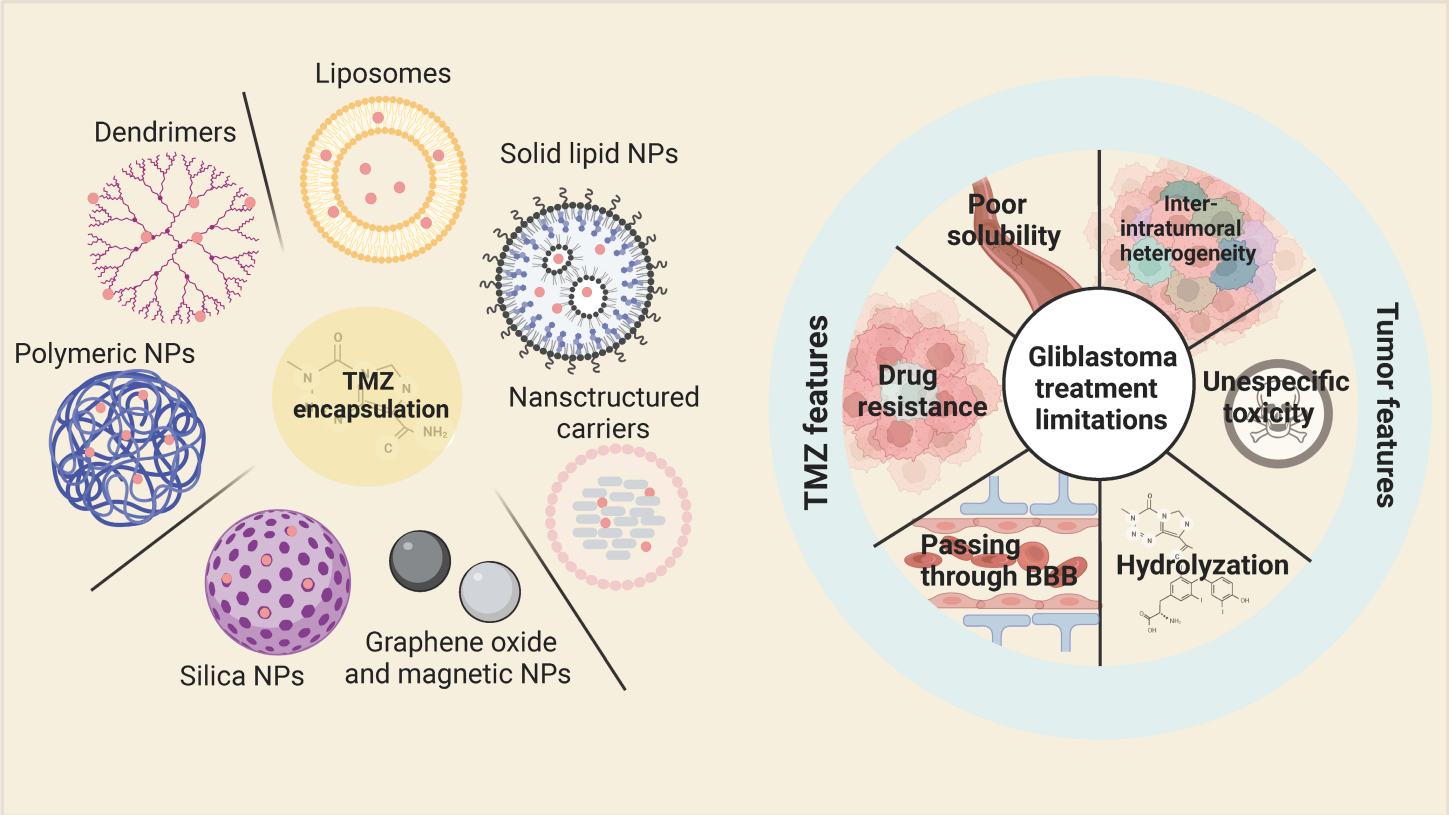
The summary of GBM treatment limitations and different possibilities for temozolomide (TMZ) encapsulation. The current limitations of TMZ for the treatment of GBM need to be overcome in order to improve its efficacy. Characteristics of TMZ include poor solubility, hydrolysis on contact with physiological media, and nonspecific toxicity. Intrinsic features of GBM include cellular and molecular heterogeneity both inter‐ and intratumorally, drug resistance, and the blood–brain barrier. The nanoparticles (NPs) utilized to package TMZ include liposomes, solid lipid NPs, nanostructured carriers, graphene oxide and magnetic NPs, silica NPs, polymeric NPs and dendrimers.

TMZ is currently the leading chemotherapeutic agent for the treatment of glioma.^66^, ^67^ TMZ is a highly bioavailable antitumor chemotherapeutic agent belonging to the imidazo‐tetrazine class. TMZ has a unique molecular structure that allows it to freely cross the BBB and enter glioma cells (GCs) to act.^68^, ^69^ TMZ can be continuously hydrolyzed to produce the active cytotoxic metabolite 5‐(3‐methyltriazen‐1‐yl)imidazole‐4‐carboxamide (MTIC). MTIC can methylate O‐6 guanine sites on GC DNA, resulting in the formation of O6 MTIC that can methylate O‐6 guanine moieties on GC DNA to form O6methylguanine, which can exert a cell killing effect on GC DNA and can perform nucleophilic attack on the DNA of GCs and exert cell‐killing effect. The high recurrence rate of gliomas and the resistance of tumor cells to TMZ are important reasons for the poor prognosis of glioma patients.^70^, ^71^ Studies have shown that various factors, including DNA repair systems, cellular autophagy, glioblastoma stem cells (GSCs), and tumor microenvironment (TME), influence GC resistance to TMZ.^72^, ^73^, ^74^ The summary of GBM treatment limitations and different possibilities for TMZ encapsulation is displayed in Figure 3.

MGMT is a DNA repair enzyme that reverses alkylating agent‐induced DNA damage by transferring a methyl group from the guanine O6 site to its cysteine residue to repair damaged O6 methylguanine nucleotides.^75^, ^76^ MGMT plays an important protective role in normal cells and its overexpression is one of the major mechanisms leading to drug resistance in GCs.^77^ However, the presence of O6 methylguanine in cells with insufficient MGMT activity results in a mismatch between guanine and thymine during DNA replication, which is recognized and repaired by the DNA mismatch repair (MMR) system, but not directly on the substrate, O6 methylguanine, resulting in persistent MMR and ultimately leads to TMZ resistance.^78^ On the other hand, DNA glycosylase and related base excision repair systems play an important role in glioma resistance to TMZ.^79^, ^80^ Cellular autophagy, the process by which the organism recycles damaged organelles and misfolded proteins through lysosomal degradation, provides stable nutrients and energy for DNA damage repair in gliomas, allows GCs to acquire invasiveness through the epithelial–mesenchymal transition pathway and to develop resistance to hypoxia and GSCs are neural stem cells derived from the subventricular zone of the brain that have unique self‐renewal and multidirectional differentiation capabilities and are drivers of GC invasion, recurrence, and drug resistance, The TME is the surrounding microenvironment in which tumor cells live and is composed of immune cells, stromal cells, extracellular matrix, and various types of signaling molecules. The dynamic interactions of the various components in the TME are essential for tumor development and progression. Compared to the normal cellular microenvironment, TME is characterized by hypoxia, low pH, high interstitial pressure, high vascular permeability, and an inflammatory response; TME is closely related to GC resistance to TMZ, and long‐term use of TMZ may exacerbate immunosuppression in glioma TME.^81^, ^82^ Angiogenic factors are particularly active in glioma TME, including transforming growth factor beta, hypoxia‐inducible factor, cyclooxygenase‐2, fibroblast growth factor, and helper T cell 2 cytokines.^83^, ^84^ Inhibition of the production of angiogenic factors, especially in glioma TME, and blocking the blood supply to the tumor are targets of action of many antiangiogenic agents.

#### 3.2.2 Other pharmaceuticals

The choice of chemotherapeutic agent for glioma should be based on a combination of the patient's unique situation and the physician's experience.^85^ Calmustine is an intravenous alkylating agent commonly used to treat low‐grade gliomas. It destroys tumor cell DNA and inhibits tumor cell division and growth.^86^ Lomustine is an orally administered alkylating agent commonly used to treat low‐grade gliomas. It inhibits the division and growth of tumor cells by destroying their DNA.^85^ Bleomycin is an antitumor agent that can be used to treat low‐grade gliomas. It can damage the DNA of tumor cells, thereby inhibiting their division and growth.^87^, ^88^ Vinblastine is an intravenously administered antineoplastic agent that can be used to treat low‐grade gliomas. It inhibits the mitogenic process of tumor cells and suppresses tumor cell growth.^89^, ^90^ These chemotherapeutic agents have certain side effects such as nausea, vomiting, hair loss, and bone marrow suppression. Therefore, the use of these agents requires careful monitoring of the patient's health status and adjustments as necessary.

## 4 OVERVIEW OF NPs

### 4.1 Physiological properties of NPs

NPs are artificially created microparticles ranging in size from 1 to 100 nm that bridge bulk materials with atoms and molecules. It has many unique properties; NPs can penetrate membrane cells and migrate along neuronal synapses, blood vessels, and lymphatic vessels.^91^, ^92^ NPs can also selectively accumulate in different cellular structures, and the continued development of NPs synthesis technology is rapidly increasing the use of NPs in drug delivery, gene delivery, bioimaging, and tumor therapy.^93^, ^94^ Nanocarriers are NPs that encapsulate a drug or have a drug adsorbed on their surface, and the strong permeability and stability of NPs greatly increases drug efficacy.^95^, ^96^ Nanoparticles (NPs), such as liposomes, are effective carriers for dissolution therapy and have been shown to significantly extend the circulating lifetime of drugs, and their long circulation time and effective penetration into the lesion site greatly increases drug tolerance and biocompatibility. The use of NPs as carriers for drug delivery has many advantages over conventional drugs, and brain‐targeted nanocarriers have been developed as materials to overcome the BBB, increase drug accumulation at brain lesion sites, and reduce toxicity to healthy brain tissue.^97^, ^98^, ^99^ Ideal nanocarriers are those that do not cleave or polymerize in the bloodstream, do not cause an immune response in the body, are targeted, and have controlled release and degradation. Nanocarriers are classified into organic and inorganic nanocarriers; organic nanocarriers include liposomes, nanocells, polymeric NPs, and dendrimers, while inorganic nanocarriers include quantum dots, magnetic nanocarriers, gold, and silver NPs.

### 4.2 Classification of nanocarriers and their applications

#### 4.2.1 Organic nanocarriers

##### Liposomes

Liposomes are lipid vesicles composed of a phospholipid bilayer and have a cell membrane‐like structure. Liposomes are biocompatible and have the ability to alter the distribution of the enclosed drug by delaying clearance and prolonging intravascular circulation time.^100^ The circulation time of liposomes is further lengthened by the inclusion of surface‐bound hydrophilic molecules such as PEG, which create a highly water‐bound barrier on the liposome surface and prevent conditioner adhesion.^101^, ^102^ PEG‐modified liposomes have a sufficiently large particle size that glomerular filtration and decrease renal excretion, thus prolonging circulation time in vivo. Liposome delivery systems are currently accepted as the most suitable delivery system for encapsulated anticancer drugs with respect to pharmacological stability and pharmacokinetics, and liposomes are biodegradable and have the potential to be loaded with high concentrations of anticancer drugs.^103^, ^104^ There are also R8 peptide‐PEGylated targeted liposomes for the treatment of gliomas, and data suggest good sustained release properties, improved stability, and increased liposomal ability to cross the blood–BBB.^105^, ^106^ Liposomes as a type of drug delivery system have significant advantages in antitumor drug delivery and are used in clinical practice to improve the efficacy of antitumor drugs and drug resistance.

##### Polymer micelles

Polymer micelles are NPs made of polymeric materials with a spherical or oval shape and size, usually tens to hundreds of nanometers in diameter.^107^, ^108^ Micelles are composed of polymer chain segments that self‐assemble to form a core and shell structure, with the core usually composed of hydrophobic polymers and the shell composed of hydrophilic polymers.^109^, ^110^ This structure gives micelles dual hydrophilic and hydrophobic properties, making them useful for a wide range of applications such as drug delivery and material preparation.^111^, ^112^ Major methods for the preparation of polymeric micelles include solvent evaporation, reversed‐phase microemulsion, and thermodynamic methods.^113^, ^114^, ^115^ Research has shown that polymeric micelles have a wide range of applications, including drug delivery, material preparation, catalysis, and oil–water separation. In drug delivery, polymeric micelles can be used as drug carriers, where drugs can be encapsulated within the micelles for targeted delivery or sustained release. In material preparation, polymeric micelles can be used to prepare nanomaterials and composites such as nanofibers and nanofilms. In catalysis, polymeric micelles can be used as catalyst supports to improve catalyst activity and selectivity. In oil–water separation, polymeric micelles can be used to adsorb and separate contaminants and toxic substances in oil and water. In conclusion, polymeric micelles are very promising nanomaterials with a wide range of applications and market prospects. Further research and improvement of preparation methods and application areas are needed in the future to better meet the needs of mankind.

##### Polymer NPs

Polymeric NPs usually consist of a polymer forming the core and a shell surrounding it.^116^, ^117^ Their surfaces can be altered by introducing surface active agents via molecules of a given different chemical structure, yielding various functional surface active agents, such as chromate and carbonate actives with antimicrobial properties.^118^, ^119^ Researchers have also found that different functions can be achieved simply by changing the size of the polymer particles.^120^, ^121^ Currently, the more studied polymers are polylactic glycolic acid (PLGA), PLA, and polyglycolic acid, which have biological properties such as long in vivo circulation time, good degradability, and low toxicity.

##### Resinous polymers

Dendrimers are unique polymer NPs whose size and morphology can be controlled during the synthesis process. Polyamide‐amine (PAMAM) dendrimers are the most widely used. They are distinguished from conventional drug delivery systems by their core–shell properties, i.e., their internal cavities can physically encapsulate small molecule drugs, while the numerous functional groups on their outer surface can complex or transport high concentrations of drug molecules.^122^, ^123^

##### DNA structure‐based origami

DNA structure‐based origami has shown potential in various fields of nanotechnology, including biomedical applications such as drug delivery systems. While DNA origami has not been extensively explored specifically for glioma treatment, it holds promise as a platform for targeted therapy and diagnosis in glioma and other cancers. In the context of glioma treatment, DNA origami can be utilized to create nanoscale carriers for targeted drug delivery. By designing the structure of DNA origami to have specific shapes and incorporating functional molecules onto its surface, researchers can potentially improve the delivery and efficacy of therapeutic agents specifically to GCs.

For instance, DNA origami structures can be functionalized with targeting ligands that selectively bind to receptors overexpressed on GCs, facilitating specific delivery of anticancer drugs or other therapeutic molecules to the tumor site. Additionally, DNA origami can be engineered to encapsulate drugs within its internal cavities, protecting them during circulation and releasing them in a controlled manner within the TME.

Furthermore, DNA origami can be employed in combination with other imaging or diagnostic modalities for glioma detection and monitoring. By incorporating imaging agents or contrast agents onto the DNA origami structure, it may be possible to enhance the visualization of glioma tumors using techniques such as magnetic resonance imaging (MRI) or positron emission tomography (PET). It is important to note that the application of DNA origami in glioma treatment is still in its early stages, and further research and development are needed to explore its potential in this specific context. However, the versatility and programmable nature of DNA origami make it an intriguing platform for targeted therapy and diagnosis, and it has already demonstrated success in other areas of nanomedicine.

#### 4.2.2 Inorganic nanocarriers

##### Graphene

Graphene nanomaterials are two‐dimensional materials composed of carbon atoms with many outstanding properties. The material's high electrical conductivity, high strength, high flexibility, and high transparency make it suitable for a wide range of applications in electronics, energy, and biomedicine.^124^, ^125^, ^126^ Graphene nanomaterials can be used to make thinner, stronger, and more transparent materials and devices, such as flexible displays, solar cells, and biosensors.^127^, ^128^, ^129^ In addition, graphene nanomaterials can be used for highly efficient electrocatalysts and battery electrode materials, as well as biomedical applications such as drug delivery and bioimaging.^130^, ^131^, ^132^ There are various methods for the preparation of graphene nanomaterials, including chemical vapor deposition, exfoliation, and reduction of graphene oxide.^133^, ^134^ Among them, chemical vapor deposition is one of the most commonly used methods to fabricate high‐quality graphene nanomaterials. In conclusion, graphene nanomaterials are two‐dimensional materials with a wide range of applications, and research on their preparation and application is constantly deepening and developing.

##### Quantum dot

Quantum dots are specialized nanomaterials with dimensions within a few nanometers. Due to their size effects, quantum dots exhibit important quantum confinement and quantum behavior.^29^, ^135^ Quantum dots have separated quantized energy spectra with wave functions that are spatially located within the quantum dot but spread over several lattice periods. Quantum dots have a small number (1–100) of integer electron, hole, and hole–electron pairs with an integer multiple of meta‐charge.^136^, ^137^, ^138^ Quantum dots have unique optoelectronic properties, allowing energy levels to be tuned by wavelength and color, and by controlling their size, particles can be made to emit or absorb light at specific wavelengths.^139^, ^140^, ^141^

##### Superparamagnetic iron oxide nanoparticles

Superparamagnetic iron oxide nanoparticles (SPIONs) are nanomaterials with superparamagnetic properties and core diameters ranging from 3 to 15 nm; SPIONs have excellent magnetic properties and stability and can be stabilized under physiological conditions.^91^, ^142^, ^143^ Such NPs can be obtained by various preparation methods, including high‐temperature thermal decomposition of organic precursors and chemical synthesis.^144^, ^145^, ^146^ Among them, high‐temperature pyrolysis of organic precursors is a commonly used preparation method to obtain SPIONs with uniform size and morphology; SPIONs can be used as contrast agents in magnetic resonance imaging to improve image resolution and contrast due to their small size and magnetic response. In addition, SPION can be used for drug delivery and magnetic thermotherapy. In drug delivery, drugs can be encapsulated in NPs and delivered precisely to the lesion site by magnetic field induction, thereby improving drug efficacy and reducing side effects.^147^, ^148^, ^149^ As for magnetic hyperthermia, the heat generated by NPs in an alternating magnetic field (AMF) can be used to kill tumor cells, thus achieving the goal of treating tumors.^150^, ^151^, ^152^ In conclusion, SPION is a promising nanomaterial with a wide range of applications, and research on its preparation and application is constantly deepening and developing.

##### Gold nanoparticles (AuNPs)

AuNPs are nanomaterials, which are gold particles with a particle size ranging from 1 to 100 nm. They have high electron density, dielectric properties, catalytic activity, and can bind to a wide range of biological macromolecules without affecting their biological activity.^153^, ^154^, ^155^ AuNPs can be prepared by chemical synthesis or biosynthesis. AuNPs have a wide range of applications in the fields of electricity, optics, and magnetism. In biology, AuNPs can be used as drug carriers to achieve targeted delivery or release of drugs by binding to drugs. They can also be used as catalysts to speed up chemical reactions and as bioimaging agents to image cells and tissues.^156^, ^157^, ^158^ Furthermore, methods for the preparation of AuNPs are constantly being improved and refined. For example, monodisperse and highly concentrated AuNPs can be prepared using seed growth methods, and the size and shape of the particles can be controlled by changing the reaction conditions.^159^, ^160^ In conclusion, AuNPs is a kind of nanomaterial with a wide range of applications, and research on their preparation and application is constantly deepening and developing.

##### Silver nanoparticles (AgNPs)

AgNPs are bacteriostatic, binding to sulfhydryl groups on the bacterial wall, blocking the bacterial respiratory chain and ultimately killing bacteria attached to the material surface.^161^, ^162^ AgNPs with a particle size of around 25 nm show strong inhibitory and killing effects against dozens of pathogenic microorganisms, including *Escherichia coli*, *Neisseria gonorrhoeae*, and *Chlamydia trachomatis*, and do not cause drug resistance.^163^, ^164^, ^165^ Cotton socks made with nanosilver and combed cotton fibers have very good antibacterial and odor control properties.

### 4.3 Current status and prospects of NPs in biomedical applications

The application of NPs in the biomedical field has gradually demonstrated their great potential and value. The use of NPs as drug carriers can deliver drugs more accurately and efficiently to lesion sites, improving drug efficacy and reducing side effects. For example, nanoliposomes and nanoemulsions have been widely used to deliver anticancer drugs.^166^ Some NPs have unique optical, magnetic, and electrical properties that can be used for bioimaging, such as iron oxide NPs for magnetic resonance imaging^167^ and carbon nanotubes for optical imaging.^168^ There are also NPs that can be used directly to treat diseases, such as gold NPs that can be used to treat cancer by killing cancer cells through heating.^109^, ^169^ Nanofibers, for example, can be used to mimic the structure of muscle and nerve tissue. In addition, nanofibers play an important role in tumor therapy, especially in overcoming the problem of tumor drug resistance.^25^, ^170^ Tumor drug resistance refers to the phenomenon in which tumor cells become resistant to chemotherapeutic drugs and is one of the main causes of tumor treatment failure. NPs as drug carriers can deliver chemotherapeutic drugs precisely to the tumor site, improving the therapeutic effect of the drug and reducing side effects.^120^, ^171^, ^172^ By designing and synthesizing various TME‐responsive NPs, novel nanocarrier systems can be constructed. These nanocarriers can deliver chemotherapeutic drugs to drug‐resistant tumor cells and overcome tumor cell drug resistance. Glioma is a current thorny issue because, in addition to its own physiology and microenvironmental biology, the BBB is also a major obstacle in the treatment of glioma disease. The BBB is composed of capillary endothelial cells, basement membranes, and astrocyte tarsal plates. Unlike other endothelial cells, the endothelial cells of the BBB are tightly packed together, forming a tight adhesive band that forms a physical barrier. This barrier also contains a number of proteins that are involved in the formation of the BBB and regulate the permeability of the BBB.^173^, ^174^, ^175^ The BBB has a protective effect on neurons, not only preventing exogenous toxins from entering the central nervous system but also preventing blocking chemotherapeutic drugs from reaching the site of the lesion, thus preventing the drugs from effectively killing tumor cells and increasing the difficulty of treatment.^176^, ^177^, ^178^ Therefore, NPs‐based drug carrier systems have been developed to overcome the limitations associated with the BBB. Binding of ligands to nanodrug delivery systems can directly target capillary endothelial cells of the BBB and facilitate internalization of nanoscale small molecule drugs via endocytosis and transcytosis.

## 5 THERAPEUTIC NPs FOR GLIOMA DRUG RESISTANCE

Studies have shown that NPs also play an important role in glioma drug resistance. The advantages and disadvantages of the different nanosystems that have been used to improve TMZ limitations based on polymers, lipids, and other nanomaterials are shown in Table 1. Zhang et al.^179^ encapsulated biocompatible PLGA‐coated TMZ and IL‐15 NPs with cRG encapsulated biocompatible PLGA‐coated TMZ and IL‐15 NPs with cRGD‐decorated NK cell membranes (R‐NKm@NPs) and designed a system of NPs that induces immunostimulatory TME and was used for chemoimmunotherapy of GBM. This system can effectively pass through the BBB and target GBM with good anti‐tumor capacity by taking advantage of the synergistic effects of the NK cell outer membrane and cRGD. Furthermore, local release of TMZ and IL‐15 after R‐NKm@NPs treatment synergistically stimulated NK cell proliferation and activation, resulting in dendritic cell maturation and CD8^+^ cytotoxic T cell infiltration. Liu et al.^180^ prepared 2‐deoxy‐d‐glucose‐modified lipopolymer NPs loaded with TMZ and si‐PD‐L1 (TMZ/siPD‐L1@GLPN/dsb) and showed that TMZ/siPD‐L1@GLPN/dsb could simultaneously deliver large amounts of TMZ and si‐PD‐L1 to TMZ‐resistant. We detected that TMZ/siPD‐L1@GLPN/dsb can simultaneously deliver large amounts of TMZ and si‐PD‐L1 to TMZ‐resistant orthotopic GBM tissues by inhibiting PD‐L1 protein expression, increasing the percentage of CD3^+^CD8^+^IFN‐γ^+^cells (Teff cells) and reducing the percentage of CD4^+^CD25^+^FoxP3^+^ cells (Treg cells) in the orthotopic TMZ‐resistant GBM tissues. T cell‐mediated cytotoxicity against orthotopic TMZ‐resistant GBM was enhanced by increasing the proportion of CD4^+^CD25^+^FoxP3^+^ cells (Treg cells) and decreasing the proportion of Treg cells. Furthermore, TMZ/siPD‐L1@GLPN/dsb also enhanced the sensitivity of in situ TMZ‐resistant GBM to TMZ by reducing the protein expression of MGMT in TMZ‐resistant GBM cells. In conclusion, TMZ/siPD‐L1@GLPN/dsb suppressed the growth of in situ TMZ‐resistant GBM and prolonged the survival of in situ GBM rats by reversing the TMZ‐resistant and immunosuppressive microenvironment. Xu et al.^181^ Temozolomide (Tem) and resveratrol (Res) were simultaneously loaded onto NPs (Tem/Res loaded mPEG‐PCL) using methoxy poly(ethylene glycol)‐poly(ε‐caprolactone) (mPEG‐PCL) as carrier and their antitumor effects were investigated. The results showed that the constructed NPs exhibited high drug loading rate and good in vitro stability; in vitro and ex vivo results showed that GCs treated with Res and Tem exhibited higher uptake efficiency and synergistic antitumor effects, as well as better tumor delay. In addition, Tem/Res‐loaded NPs had a greater ability to induce apoptosis than those treated with the free drug combination; Tem/Res co‐polymerized particles more effectively inhibited phosphorylated Akt, leading to up‐regulation of downstream apoptotic proteins mechanism experiments showed that Tem/Res copolymerized particles more effectively inhibited phosphorylated Akt, leading to upregulation of downstream apoptotic proteins. Stephen et al.^182^ developed superparamagnetic iron oxide NPs ([NPCP‐BG‐CTX]) consisting of a magnetic core and a redox‐reactive crosslinked biocompatible chitosan‐PEG copolymer surface coating (NPCP), which were used to target benzylguanine to GBM for convection enhanced delivery. Covalent attachment of a chlorotoxin (CTX)‐targeted peptide‐modified NPCP enhanced delivery of benzylguanine to GBM; cells treated with NPCP‐BG‐CTX showed significantly reduced MGMT activity and enhanced TMZ toxicity. In conclusion, their experimental results confirmed that NPCP‐BG‐CTX has good physicochemical properties, tumor cell‐specific BG delivery, controlled BG release, and better in vivo efficacy. Kim et al.^183^ developed a systemic nanodelivery platform (scL) for tumor‐specific targeting. scL was found to cross the BBB and effectively target GBM and cancer stem cells. Furthermore, systemic administration of scL‐p53 downregulated MGMT and induced apoptosis in intracranial GBM xenografts. On the other hand, the combination of scL‐p53 and TMZ significantly improved the antitumor effect of TMZ and the survival rate of TMZ‐highly resistant GBM mouse models. Zeng et al.^184^ designed an all‐in‐one therapeutic nanoprobe (PEG/αCD25‐Cy7/TMZ) for real‐time immune response tracking for precision chemotherapy and photoacoustic fluorescence imaging of GBM. In this nanoprobe, TMZ and αCD25‐Cy7, an optical dye that targets regulatory T lymphocytes, were encapsulated by glutathione‐responsive DSPE‐SS‐PEG2000 to increase the targeting efficiency of regulatory T lymphocytes, and glutathione‐induced PEG/αCD25‐Cy 7/TMZ activation enhanced TMZ delivery to the TME. Accurate delivery and monitoring of glioblastoma boundaries by photoacoustic fluorescence imaging. Immunotherapy with an indoleamine‐2,3‐dioxygenase inhibitor after chemotherapy significantly enhanced immune responses, reduced regulatory T lymphocyte infiltration, and improved survival. In conclusion, nanoprobes are capable of both accurate delivery of TMZ and long‐term dynamic tracking of regulatory T lymphocytes for accurate tumor chemoimmunotherapy.

**TABLE 1:** The advantages and disadvantages of the different nanosystems that have been used to improve TMZ limitations based on polymers, lipids, and other nanomaterials.

Types	Advantages	Disadvantages
*Polymer‐based nanosystems*		
Polymeric nanoparticles	It has a very high surface area and volume ratio, which enables more effective material transfer and reaction, improving energy utilization and efficiency. They can be designed according to requirements including shape, size, and composition, which makes them highly adaptable in various applications. It has good physical and chemical stability and can maintain its performance under a wide range of temperature and humidity conditions. It has good biocompatibility and low toxicity and can be used for biomedical applications such as drug delivery tissue engineering, and biological imaging	The preparation process usually requires precise instruments and conditions, resulting in high production costs. May cause pollution to the environment, such as microplastic pollution. It may trigger an immune response, especially if it exists in the body for a long time. There are still many unknown factors and uncertainties regarding the performance and safety of certain polymer nanoparticles, which require further research and exploration
Dendrimers	Has high elasticity and toughness. Good chemical stability, able to resist chemical corrosion. Good conductivity and optoelectronic performance, suitable for use in electronic devices. Easy to functionalize and modify, it can be used for drug delivery and biomedical applications, such as drug delivery, gene therapy, and biological imaging	The synthesis process is relatively complex and requires the use of multiple raw materials and catalysts. The unevenness of the structure may lead to unstable performance. Compared to linear polymers, dendritic polymers have poorer solubility
*Lipid‐based nanosystems*		
Liposomes	It can serve as a drug delivery carrier, can be modified with various ligands and functional molecules, and can be used for targeted delivery of drugs and gene drugs. Good organizational compatibility. Can reduce the toxicity of drugs to the heart and kidneys. Long circulating liposomes can prolong the retention time of drugs in the blood, which is beneficial for enhancing drug efficacy. The drug loading range is wide. There are various routes of administration	High preparation cost, inter batch differences, sterilization requirements, increased encapsulation efficiency, particle size control, short shelf life of liposomes, and potential issues with residual organic solvents
Solid lipid nanoparticles	It can efficiently deliver drugs to the target site, improve drug bioavailability and efficacy, have good biocompatibility, and stable physical properties	The complex preparation process may cause adverse reactions, and factors such as the chemical properties and molecular weight of drugs can affect their compatibility and stability with nanoparticles
Nanostructured carriers	It can efficiently carry drugs, increase drug concentration and distribution in the body, and improve drug efficacy. Targeted drug delivery can be achieved through surface modification or internal drug loading, improving drug targeting and efficacy. It can control the release rate of drugs, achieve sustained release of drugs, and reduce the toxic side effects and adverse reactions of drugs. It can reduce the dosage and concentration of drugs, thereby reducing their toxic side effects and adverse reactions. Modification or treatment can improve its biocompatibility, reduce immune and inflammatory responses in the body	High preparation complexity, high cost, limited biodegradability, slow degradation rate in vivo, may affect drug release and efficacy, and may have potential toxic effects on the body, such as causing immune and inflammatory reactions. Insufficient stability in the body may be recognized and attacked by the body's immune system, leading to unstable or uneven drug release
*Other nanomaterials*		
Mesoporous silica nanoparticles	It has excellent biocompatibility and can stably exist in the in vivo environment without causing serious immune or inflammatory reactions. It has a large specific surface area and pore volume, and can effectively adsorb various small molecule substances. Has excellent chemical stability and can maintain stability under various environmental conditions	High production cost, difficult to degrade, and poor consistency between batches
Graphene oxide nanoparticles	Good biocompatibility, excellent chemical stability, good electrochemical performance, and easy functionalization modification	The preparation process is complex, difficult to control particle size and morphology, and may cause environmental pollution
Magnetic nanoparticles	Highly customizable, highly magnetic responsive, excellent biocompatibility, and easy in vivo tracking	High production cost, potential toxicity, and various factors may affect its structure and performance in the internal environment, affecting its application effectiveness. Its ability to penetrate tissues is limited, making targeted delivery to deep tissues more difficult

Furthermore, NPs can bind to other drugs and thus play an important role in the treatment of gliomas. Di Mascolo et al.^185^ fabricated a conformal polymer implant, μMESH, by sandwiching PLGA‐edged micromesh on a polyvinyl alcohol (PVA) column array and applied it to the sustained delivery of the potent chemotherapeutic molecules docetaxel (DTXL) and paclitaxel (PTXL) were applied for sustained delivery. Four types of μMESH were designed with DTXL or PTXL encapsulated in PLGA micronets and DTXL (nano‐DTXL) or PTXL (nano‐PTXL) nano‐sized in PVA microlayers. Sandbhor et al.^186^ prepared lipid nanoparticles (LNPs) and used them to coadminister transferrin‐modified PTX and the proapoptotic drug miltefosine (HePc) (Tf‐PTX‐LNPs). The results showed that the dual‐drug anti‐glioma effect of lipid‐targeted alternative delivery NP systems (Tf‐PTX‐LNPs) of transferrin receptor (TfR)‐based PTX and the proapoptotic drug HePc significantly overcame O6‐methylguanine‐DNA methyltransferase‐induced resistance, and improve the therapeutic efficacy of GBM. Furthermore, the prepared intranasal target LNPs were biocompatible and stable, had high BBB transporter capacity, and could effectively penetrate GBM‐induced mouse brain tumors. Furthermore, in vivo administration of GBM‐targeted LNPs significantly prolonged survival and improved antitumor effects in cancer‐bearing mice compared to systemically administered Taxol® or intranasally administered free drug, and reduced drug toxicity. Kang et al.^187^ loaded Dp44mT onto PEGylated PLGA NPs modified with the glioma‐targeting ligand interleukin 13 (IL13). IL13 modification significantly enhanced NP uptake by GCs and improved transport in an in vitro BBB model The IL13 modification significantly enhanced NP uptake by GCs and improved transport in an in vitro BBB model. When tested, this targeted agent showed superior toxicity against GC lines and patient stem cells in vitro and did not cause significant killing of healthy brain microvascular endothelial cells. In addition, in vivo results showed that IL13‐bound Dp44mT‐NPs significantly inhibited glioma tumor growth and did not cause significant weight loss or renal/hepatic toxicity in mice. Kinoh et al.^188^ applied pH‐sensitive epirubicin micellar nanomedicine to synergize the effects of anti‐PD1 antibody (aPD1) on PTEN‐positive and PTEN‐negative orthotopic GBMs. The results showed that pi/m and aPD1 transformed cold GBM into hot tumors highly infiltrated with anti‐tumor immune cells by inducing immunogenic cell death, eliminating immunosuppressive myeloid‐derived suppressor cells and reducing PD‐L1 expression on tumor cells. Chen et al.^189^ showed that GBM cell (GBC)‐derived IL‐6 upregulates IL‐6 expression on astrocytes by activating STAT3. We found that astrocyte‐derived IL‐6 reacts with GBCs to further activate STAT3 and promote the proliferative, migratory, invasive, and antiapoptotic abilities of GBCs. We also found that doxorubicin‐polyglycerol‐nanodiamond conjugate (nanoDOX) inhibits STAT3 activity of GBCs via GBM‐associated macrophage delivery and reduces IL‐6 export from GBCs to astrocytes, thus reducing astrocyte‐induced GBC We also found that feedback activation can be eliminated. Furthermore, NanoDOX was able to inhibit the stimulated activation of STAT3 and IL‐6 induced by TMZ, thereby suppressing drug resistance. Li et al.^190^ showed that Nano‐DOX‐loaded TAMs are active and can infiltrate 3D GC spheroids to release drugs and that GCs can induce Nano‐DOX‐loaded TAMs to return Nano‐DOX to GCs and release damage associated molecular patterns (DAMP). Furthermore, Nano‐DOX induced the release of DAMPs in GCs more than doxorubicin, and nano‐DOX‐damaged GCs reprogrammed TAMs from a GBM‐supportive phenotype to an anti‐GBM phenotype that inhibited GC growth In vivo experiments showed that Nano‐DOX can effectively inhibit tumor progression inhibit tumor progression. Zhang et al.^191^ developed a strategy to promote nano‐encapsulation of arsenic trioxide (ATO) with manganese (Mn). Experimental results showed that the formation of arsenite (As(3+))‐Mn precipitates in liposomes produced a magnetic induction effect, while cellular uptake degraded the As‐Mn complex, releasing ionic As(3+) and Mn(2+) at low pH in the presence of endogenous liposomes. They also showed that the targeted nano‐formulation ATO can effectively treat TMZ‐resistant GBM and that a convertible manganese contrast agent is an alternative to clarify the ability of ATO delivery and release. Tao et al.^192^ synthesized a paclitaxel‐loaded nanopreparation (nano‐PTX) and investigated its absorption, release, and toxicological properties. The results showed that treatment of U87 cytoblasts with bone marrow‐derived macrophage (BMM)‐nano‐PTX significantly increased chemosensitivity and inhibited cell survival compared to nano‐PTX alone. In conclusion, PTX nano‐formulations enhance cellular uptake, delay toxicity, and increase efficacy with BMM‐nano‐PTX delivery. Kim et al.^183^ conjugated DOX to poly(ethylene glycol) (PEG) via pH‐sensitive stilbene bonding to poly(β‐l‐malic acid) (PMLA), a biodegradable, non‐toxic, non‐immunogenic nanoconjugate platform. The DOX nanoconjugate was found to be physiologically The DOX nanoconjugates were found to be highly stable under physiological conditions and significantly inhibited cancer cell growth in vitro in a primary GC line. Beola et al.^193^ established multifunctional lipid‐magnetic nanocarriers (Ang‐TMZ‐LMNVs) with vascular endothelial peptide‐2 function and loaded with TMZ and evaluated their value in GBM therapy. The results showed that Ang‐TMZ‐LMNVs were able to accumulate and remain in tumors after local administration, and in combination with AMFs stimulation effectively suppressed tumor invasion and growth, and significantly prolonged median survival. Li et al.^194^ synthesized carmustine (BCNU)‐loaded hypoxia‐responsive nanomicelles (T80‐HA‐AZO‐BG/BCNU NPs) with BBB permeability and O6‐alkylguanine‐DNA alkyltransferase (AGT) inhibitory activity. The synthesized T80‐HA‐AZO‐BG/BCNU NPs exhibited stability, biocompatibility, and hypoxia‐responsive drug release ability, and T80 modification improved micelle transport in an in vitro BBB model. Furthermore, the T80‐HA‐AZO‐BG/BCNU NPs pair significantly enhanced the cytotoxicity of GC lines with high AGT expression compared to the conventional BCNU and O6‐BG combination.

## 6 DISCUSSION AND PROSPECTS

Gliomas are the most common cranial malignant brain tumors in neurosurgery and are not curable; GBMs are the most aggressive tumors and generally require postoperative treatment with radiation therapy and chemotherapy. However, patient prognosis remains very poor, with most patients having a short survival time even with regular treatment. Complete resection remains difficult because of the proliferative and invasive nature of the disease. On the other hand, malignant gliomas have acquired resistance to chemotherapeutic agents such as TMZ, which is another reason why treatment does not yield the expected results. At present, tumor cells are relatively tolerant to chemotherapy, so further research is needed to find new chemotherapeutic options for the tumors.

Nanomaterials have unparalleled advantages and potential in drug‐targeted transport applications because of their versatility, their raw materials are relatively inexpensive and readily available as carriers for antitumor drug‐targeted therapy, their long circulation time in the body, and their relatively low toxicity. Therefore, NPs have broader prospects and application value in the delivery of antitumor and antiviral drugs, antigens and vaccines. It can be used to achieve targeted delivery of drugs through surface modification and structural design to more precisely deliver drugs to lesion sites. In addition, NPs can improve the bioavailability of drugs by increasing their solubility and stability and avoiding their rapid degradation and excretion in vivo. NPs can also improve drug efficacy and reduce side effects through targeted drug delivery. Targeted drug delivery can reduce the distribution and dosage of drugs in normal tissues, thereby reducing side effects. However, targeted drug delivery by NPs has its challenges. Because of the large surface area of NPs, water molecules and other molecules adsorbed on the surface tend to cause NPs to aggregate, reducing their stability. Because receptors on the surface of different tumor and lesion cells are not the same, researchers need to develop NPs that can target multiple receptors simultaneously to improve therapeutic efficacy against different diseased tissues. Currently, the cost of manufacturing NPs is high, and further development of low‐cost and efficient preparation methods is needed to facilitate the application of NPs for targeted drug delivery. The biocompatibility and safety of NPs need to be further investigated and verified to ensure that they do not adversely affect normal tissues.

Drug resistance is a phenomenon in which tumor cells simultaneously become resistant to drugs with different structures and mechanisms of action and is one of the main causes of failure of tumor therapy, achieved through the design of specialized NPs that encapsulate various drug molecules inside the particle and simultaneously achieve controlled release and targeted delivery of the drug. Controlled release and targeted delivery. Such NPs can carry drugs simultaneously and increase the exposure of tumor cells to the drug, thus enhancing therapeutic efficacy and reducing the emergence of drug resistance. In terms of drug selection, chemotherapeutic drug encapsulation or combination therapy can be selected for different tumor cell types and drug resistance mechanisms. Targeted delivery of NPs can reduce the distribution and dosage of drugs in normal tissues, thus reducing drug damage to normal tissues and the occurrence of side effects. In addition, specialized NPs can be designed that can overcome the problem of multidrug resistance in tumor cells, improve therapeutic efficacy, and prolong patient survival. However, there are still several challenges and problems in reversing drug resistance in NPs. First, the cost of preparing and manufacturing NPs is high, and further development of low‐cost and efficient preparation methods is needed. The biodistribution and pharmacokinetic behavior of NPs need to be further studied and optimized to ensure effective concentration and duration of drug action in diseased tissues. In addition, the safety and long‐term effects of NPs need to be further evaluated and validated to ensure that NPs do not adversely affect normal tissues. In conclusion, drug resistance reversal of NPs is a promising therapeutic strategy to overcome the problem of drug resistance in tumor cells, improve therapeutic efficacy, and prolong patient survival. However, further studies and validation are needed to ensure its safety and efficacy.

Glioma is a common intracranial tumor and its treatment has always been a challenge for the medical community. In recent years, NPs as a new material have shown great potential in the treatment of gliomas due to their unique properties. As a kind of drug carrier, NPs have high drug solubility and permeability, which can effectively improve drug efficacy and reduce side effects. By encapsulating drugs in NPs, precise control and targeted release of drugs can be achieved, increasing drug concentrations in tumor tissues and improving therapeutic efficacy. In addition, NPs can further enhance the anti‐tumor effects of drugs by facilitating cellular uptake and improving intracellular distribution. Targeting is an important property of NPs in glioma therapy. Through surface modification and ligand recognition, NPs can achieve targeted delivery to tumor tissue and local release. This targeting effect can reduce drug damage to normal tissues and improve therapeutic efficacy. Furthermore, the targeting effect can further enhance therapeutic efficacy by increasing drug concentrations in tumor tissue and inducing apoptotic pathways in tumor cells. In addition to direct pharmacotherapy, it can also be used to treat gliomas by modulating the immune response. Furthermore, NPs can act as immunomodulators to suppress the immune escape of tumor cells and enhance the anti‐tumor effect of the organism. First, the safety of NPs needs to be further evaluated to ensure their safety in clinical applications. Second, the preparation and production of NPs need to be standardized and scaled up to meet clinical needs. Finally, the use of NPs in combination with other therapeutic approaches needs to be further investigated to enhance therapeutic efficacy and improve patient quality of life.

## 7 CONCLUSION

With the advent of NPs and the widespread use of nanotechnology in clinical practice, NPs‐based drugs in tumor therapy have gradually gained more attention and application by clinical practitioners. This article outlines and summarizes the biological properties, types, and current use of NPs in glioma chemotherapeutic agents, as well as the prospects for NPs research and development in recent years.

## AUTHOR CONTRIBUTIONS

Jiyuan Liu and Xiuchun Zhang: Original draft preparation, allocation. Jinqu Hu and Fan Yang: revision, supplement and edition. All authors have read and agreed to the published version of the manuscript.

## FUNDING INFORMATION

None.

## CONFLICT OF INTEREST STATEMENT

The authors declare no conflict of interest.

## Data Availability

The data that support the findings of this study are available in the supplementary material of this article.
